# Characteristics of the inner retinal layer in the fellow eyes of patients with unilateral exudative age-related macular degeneration

**DOI:** 10.1371/journal.pone.0239555

**Published:** 2020-09-23

**Authors:** Seong Eun Lee, Hyung Bin Lim, Yong Il Shin, Cheon Kuk Ryu, Woo Hyuk Lee, Jung-Yeul Kim

**Affiliations:** 1 Department of Ophthalmology, Chungnam National University College of Medicine, Daejeon, Republic of Korea; 2 Rhee’s Eye Hospital, Daejeon, Republic of Korea; Massachusetts Eye & Ear Infirmary, Harvard Medical School, UNITED STATES

## Abstract

**Objective:**

To investigate the thicknesses of the ganglion cell-inner plexiform layer (GC-IPL) and retinal nerve fiber layer (RNFL) of the fellow eyes of patients with unilateral exudative age-related macular degeneration (AMD).

**Methods:**

A total of 107 patients with unilateral exudative AMD [34 of typical choroidal neovascularization (tCNV), Group A; 73 of polypoidal choroidal vasculopathy (PCV), Group B] and 73 normal control eyes (Group C) were included. Drusen and subretinal drusenoid deposits were assessed in all participants using fundus photography, autofluorescence, and optical coherence tomography (OCT). The GC-IPL and RNFL thicknesses were measured using Cirrus HD-OCT and compared among groups. Linear regression analyses were used to evaluate the factors associated with GC-IPL thicknesses.

**Results:**

The average GC-IPL thicknesses of Groups A, B, and C were 77.09 ± 3.87, 80.10 ± 6.61, and 80.88 ± 6.50 μm, respectively (p = 0.022). Sectoral GC-IPLs and central macular thicknesses (CMTs) were significantly different among groups (all, p <0.05), whereas none of the RNFL parameters differed significantly (all, p >0.05). Multivariate linear regression analyses revealed that age (p <0.001), CMT (p <0.001), and tCNV (p = 0.013) were significantly associated with average GC-IPL thickness, and the rate of reduction of GC-IPL thickness with increasing age in the fellow eyes of tCNV patients was higher than those in the PCV and control groups.

**Conclusions:**

Unilateral tCNV patients exhibited statistically significant reduction of the GC-IPL thickness in the fellow eyes, compared to values of the fellow eyes of unilateral PCV patients or control patients. RNFL values trended to be lower but did not reach statistical significance.

## Introduction

Age-related macular degeneration (AMD) is a leading cause of irreversible blindness in elderly people in industrialized countries, and the third-most common cause of blindness worldwide [1, 2]. Regarding subtypes of neovascular AMD, occult choroidal neovascularization is the most common in Western populations [3], whereas polypoidal choroidal vasculopathy, which is a variant of neovascular AMD, is the predominant form in Asian populations [2, 4]. Although these subtypes share some clinical features, they have different epidemiological and pathophysiological characteristics, as well as different treatment outcomes.

Although it is well-known that the outer retinal layers are mainly affected in AMD, several studies have reported that thinning of the inner retinal layers, including the ganglion cell-inner plexiform layer (GC-IPL) and retinal nerve fiber layer (RNFL), are also observed in dry type [5–7] and exudative AMD [8, 9]. Several mechanisms have been recently introduced to explain the damage observed in the inner retinal layers, such as transneuronal degeneration or chronic ischemia [10–12]. In addition, it has also been reported that the ganglion cell layer thickness decreases as AMD progresses [8]. These findings suggest that AMD is a risk factor that should be considered in the analysis of the inner retinal layer.

In many cases, bilateral involvement of exudative AMD was observed, and it was reported that approximately 43% of unilateral exudative AMD patients develop typical fellow eye choroidal neovascularization (tCNV) within 5 years [13]. In addition, Baek et al. also reported that 84% of the fellow eyes of unilateral polypoidal choroidal vasculopathy (PCV) or aneurysmal type 1 neovascularization patients have outer retinal abnormalities [14]. Considering these results, analyses of the fellow eyes of unilateral exudative AMD patients could provide important clues relevant to the analyses of AMD progression and its clinical characteristics. In the present study, we therefore assessed the inner retinal layer characteristics of the fellow eyes in unilateral exudative AMD patients. Our aim was to compare thicknesses of the inner retinal layers, including the GC-IPL and RNFL, among subtypes of exudative AMD patients, to identify factors associated with inner retinal layer damage.

## Methods

This was a retrospective, observational, comparative study. The study protocol was approved by the institutional review board of Chungnam National University Hospital (Daejeon, Republic of Korea) and adhered to the tenets of the Declaration of Helsinki.

### Participants

**Fig 1:**
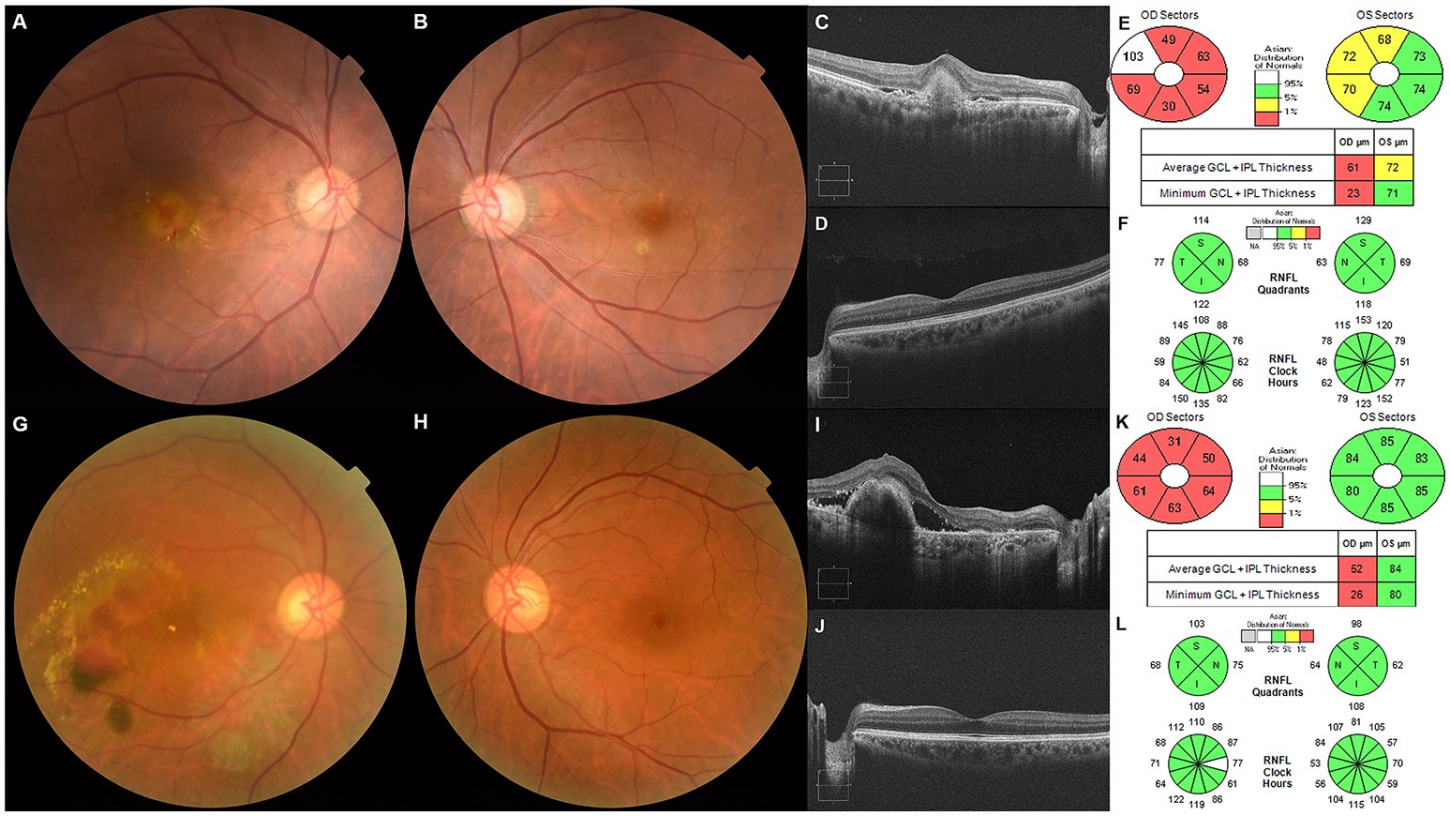
Fundus photography (A, B, G, H), optical coherence tomography images including line scan (C, D, I, J), ganglion cell—inner plexiform layer (GC-IPL) analysis map (E and K), and retinal nerve fiber layer (RNFL) analysis map (F and L) of representative cases showing unilateral exudative age-related macular degeneration. Typical choroidal neovascularization (tCNV) was observed in the right eye (A and C), and drusen and the focal depigmented lesion were noted in the left eye (B and D). The left eye (G and I) was diagnosed with polypoidal choroidal vasculopathy (PCV), and there was no abnormal finding in the right eye (H and J). The average GC-IPL and RNFL thickness was 72μm and 95μm in the fellow eye with tCNV, and 84μm and 97μm in the fellow eye with PCV, respectively.

This study included patients with treatment-naive unilateral tCNV and PCV who visited the Chungnam National University Hospital retinal clinic from June 2016 to December 2018. Patients who met the inclusion and exclusion criteria were consecutively included. Patients with unilateral tCNV were placed in Group A (Fig 1A–1F), and patients with unilateral PCV were placed in Group B (Fig 1G–1L). Age- and sex-matched normal subjects were placed in Group C (control). Exudative typical CNV was diagnosed when there was evidence of CNV associated with nondrusenoid retinal pigment epithelium (RPE) detachment, serous or hemorrhagic retinal detachment, subretinal hemorrhage, or subretinal exudation [15]. Inclusion criteria for PCV in this study were based on the EVEREST study criteria-the presence of early subretinal hyperfluorescent lesions upon analysis using indocyanine green angiography (ICGA) and other features including the nodular appearance of polyps when viewed stereoscopically, hypofluorescent halos around the nodule, pulsatile filling of polyps, branching vascular networks, and an orange appearance of nodules corresponding to ICGA lesions upon color imaging [16].

Comprehensive ophthalmic examinations, including slit-lamp microscopy; fundus examination; and measurements of uncorrected visual acuity, best-corrected visual acuity (BCVA) using the Snellen chart, refraction using an automatic refractometer, and intraocular pressure (IOP) and axial length (AXL) using an IOL Master (Carl Zeiss, Jena, Germany); fundus photography; fluorescein angiography (FA) and ICGA using the Heidelberg Retina Angiograph 2 (Heidelberg Engineering, Heidelberg, Germany); and optical coherence tomography (OCT; Carl Zeiss Meditec, Dublin, CA, USA), were performed. All fundus photographs and FA, ICGA and OCT images were reviewed by two independent investigators (H.B.L. and S.E.L.) to categorize them into groups. If there was any disagreement, a senior investigator (J.Y.K.) was invited for discussion until a consensus was reached.

Patients presenting only unilateral tCNV or PCV were included in the study. Patients with conditions that could affect the RPE and choroid status of both eyes (e.g., history of systemic steroid use and other concurrent diseases such as diabetic retinopathy, hypertensive retinopathy, uveitis, or tumors) or those with a history of anti-vascular endothelial growth factor treatment, prior laser or photodynamic therapy, or retina or choroid trauma were excluded. Patients with other ocular diseases of either eye that could affect the GC-IPL thickness, such as glaucoma, or retinal or neuro-ophthalmic diseases, or those treated with intraocular surgery other than cataract surgery, were excluded. The fellow eyes with CMT <200 μm were considered as having macular atrophy [17, 18] and were excluded from the study. The fellow eyes with BCVA <20/25 or high myopia (AXL ≥26.0 mm or spherical equivalent ≤ –6.0 diopters) were also excluded. Normal subjects (Group C)) were also enrolled, and they had no history of ocular disease, normal anterior segment, BCVA ≥ 20/25, IOP in the normal range, and a spherical equivalent within ± 3.0 D.

The presence of soft drusen, pachydrusen, and subretinal drusenoid deposits (SDD) was evaluated using fundus photography, autofluorescence, and OCT. The definition of soft drusen and SDD followed that described in a previous report [19]. Pachydrusen were considered present if there were isolated or scattered yellowish-white deposits on fundus photography that corresponded to the presence of homogenous material accumulation under RPE on OCT images [20].

### OCT

The OCT parameters were measured by an experienced examiner with a 512 × 128 macular cube and a 200 × 200 optic cube scanning protocol using a Cirrus HD-OCT instrument (Carl Zeiss Meditec, Dublin, CA, USA). The macular cube scan was assessed using ganglion cell analysis, which automatically measured the GC-IPL thickness by identifying the layer between the outer boundaries of the RNFL and the inner plexiform layer in three dimensions. The elliptical annulus was defined by the vertical radii (outer radius and inner radius of 2.0 and 0.5 mm, respectively) and horizontal radii (outer radius and inner radius of 2.4 and 0.6 mm, respectively) The average, minimum, and six sectoral (superior, superotemporal, superonasal, inferior, inferonasal, and inferotemporal) GC-IPL thicknesses were measured. The CMT was measured using retinal map analysis. The average and four sectoral (superior, temporal, inferior, and nasal) RNFL thicknesses were also evaluated. Optic nerve head parameters, such as the rim area, disc area, average cup/disc ratio, and cup volume were measured as well. The CMT and GC-IPL thicknesses were measured with the image centered on the fovea. Two researchers (H.B.L. and S.E.L.) reviewed the image, and if there were problems such as signal strength < 7, segmentation error, motion artifact or misalignment, they were excluded from the study.

### Statistical analyses

Statistical analyses were performed using SPSS statistical software for Windows (ver. 21.0; IBM Corporation, Armonk, NY, USA). The GC-IPL, central macula, and RNFL thicknesses were compared among the three groups using analysis of variance, followed by a post hoc test (the Bonferroni test). The chi-square test and Fisher’s exact test was used to compare categorical data. Analyses of covariance (ANCOVA) were also used to control the effects of covariate values such as sex, CMT, disc area, and cup volume. Univariate and multivariate linear regression analyses were used to evaluate the factors affecting GC-IPL thickness. In all analyses, a value of p <0.05 was considered statistically significant.

## Results

### Patient demographics

This study enrolled a total of 180 subjects, including 34 tCNV patients (Group A), 73 PCV patients (Group B), and 73 control subjects (Group C). The mean ages in Groups A, B, and C were 70.74 ± 8.70, 69.18 ± 7.86, and 70.60 ± 7.54 years, respectively (p = 0.473; Table 1). The proportion of female patients in Group B was lower than those of the other groups (p = 0.019). Histories of diabetes and hypertension, BCVA, spherical equivalents, IOP, and AXL did not differ significantly among groups (all, p >0.05). There were no significant differences in rim area (p = 0.417) and cup/disc ratio (p = 0.201), whereas disc area (p = 0.003) and cup volume (p = 0.008) were significantly different among groups. The mean CMTs in Groups A–C were 246.03 ± 18.09, 253.30 ± 22.31, and 257.11 ± 20.26 μm, respectively (p = 0.039), and the mean value for Group A was significantly lower than that for Group C (p = 0.033, post hoc test). Soft drusen and SDD were found more frequently in Group A (soft drusen: 82.4%, p <0.001; SDD: 29.4%, p = 0.004) than in Group B (soft drusen: 6.8%, SDD: 4.1%). However, pachydrusen were more common in Group B (21 eyes; 28.8%, p = 0.015) than in Group A (2 eyes; 5.9%).

**Table 1:** Demographic and clinical characteristics of the study subjects.

	Group A (n = 34)	Group B (n = 73)	Group C (n = 73)	p-value	p-value^a^	p-value^b^	p-value^c^
Age (mean ± SD; years)	70.74 ± 8.70	69.18 ± 7.86	70.60 ± 7.54	0.473*	1.000	1.000	0.832
Sex ratio (male/female)	16/18	52/21	38/35	**0.019**^†^			
Diabetes (n, %)	5 (14.7)	10 (13.7)	13 (17.8)	0.782^‡^			
Hypertension (n, %)	14 (41.2)	31 (42.5)	35 (47.9)	0.732^†^			
BCVA (mean ± SD; logMAR)	–0.02 ± 0.04	–0.01 ± 0.03	–0.02 ± 0.04	0.092*	0.428	1.000	0.115
Spherical equivalent (mean ± SD; diopters)	–0.10 ± 1.17	0.23 ± 1.05	0.52 ± 2.51	0.241*	1.000	0.297	0.993
Intraocular pressure (mean ± SD; mmHg)	14.85 ± 2.68	14.23 ± 2.46	14.38 ± 2.59	0.512*	0.752	1.000	1.000
Axial length (mean ± SD; mm)	23.57 ± 1.06	23.62 ± 0.85	23.69 ± 0.92	0.804*	1.000	1.000	1.000
Rim area (mean ± SD; mm^2^)	1.31 ± 0.25	1.25 ± 0.27	1.25 ± 0.22	0.417*	0.674	0.677	1.000
Disc area (mean ± SD; mm^2^)	1.92 ± 0.37	2.00 ± 0.38	1.81 ± 0.27	0.003*	0.848	0.286	**0.002**
Cup/disc ratio (mean ± SD)	0.53 ± 0.16	0.57 ± 0.16	0.52 ± 0.15	0.201*	0.606	1.000	0.290
Cup volume (mean ± SD; mm^3^)	0.14 ± 0.12	0.22 ± 0.17	0.15 ± 0.16	0.008*	**0.048**	1.000	**0.016**
Central retinal thickness (mean ± SD; μm)	246.03 ± 18.09	253.30 ± 22.31	257.11 ± 20.26	0.039*	0.280	**0.033**	0.807
Soft drusen (n, %)	28 (82.4)	5 (6.8)	N.A.	**<0.001**^‡^			
Pachydrusen (n, %)	2 (5.9)	21 (28.8)	N.A.	**0.015**^‡^			
Subretinal drusenoid deposit (n, %)	10 (29.4)	3 (4.1)	N.A.	**0.004**^‡^			

Group A, fellow eyes of choroidal neovascularization patients; Group B, fellow eyes of polypoidal choroidal vasculopathy patients; Group C, control group; SD, standard deviation; BCVA, best-corrected visual acuity; logMAR, logarithm of the minimum angle of resolution; N.A., not applicable.

Values in boldface are statistically significant (p <0.05).

*P-value from one-way analysis of variance among the three groups followed by post hoc multiple comparison.

^†^P-value from the chi-square test.

^‡^P-value from Fisher’s exact test.

^a^P-value from post hoc test (Bonferroni) between Groups A and B.

^b^P-value from post hoc test (Bonferroni) between Groups A and C.

^c^P-value from post hoc test (Bonferroni) between Groups B and C.

### Comparison of GC-IPL and RNFL thicknesses

The average GC-IPL thicknesses in Groups A–C were 77.09 ± 6.87, 80.10 ± 6.61, and 80.88 ± 6.50 μm, respectively. The average and all subfields of GC-IPL thickness differed significantly among the three groups (all, p <0.05). In the post hoc analyses, all GC-IPL measurements in Group A were significantly lower than those in Group C (all, p <0.05, Table 2), and no statistically significant differences were observed for all GC-IPL measurements between groups A and B and between groups B and C. For the RNFL, no significant differences were observed for the average and all sectors among all groups both in ANOVA and post hoc analyses (p >0.05).

**Table 2:** Comparison of ganglion cell-inner plexiform layer and retinal nerve fiber layer thicknesses among Groups A–C.

		Group A (n = 34) (mean ± SD; μm)	Group B (n = 73) (mean ± SD; μm)	Group C (n = 73) (mean ± SD; μm)	p-value*	p-value^†^	p-value^‡^	p-value^§^
GC-IPL	Average	77.09 ± 6.87	80.10 ± 6.61	80.88 ± 6.50	**0.022**	0.089	**0.019**	1.000
	Minimum	71.21 ± 11.83	75.26 ± 11.65	76.67 ± 8.24	**0.043**	0.189	**0.038**	1.000
	Superior	75.09 ± 9.64	78.44 ± 7.02	80.68 ± 7.76	**0.002**	0.104	**0.002**	1.000
	Superotemporal	76.47 ± 7.46	79.96 ± 7.95	79.82 ± 5.91	**0.043**	0.057	**0.072**	1.000
	Inferotemporal	77.91 ± 5.81	81.29 ± 7.23	81.90 ± 6.71	**0.016**	0.052	**0.015**	1.000
	Inferior	75.12 ± 6.85	78.00 ± 7.32	78.96 ± 7.14	**0.037**	0.162	**0.032**	1.000
	Inferonasal	76.65 ± 6.46	78.64 ± 8.43	80.67 ± 7.80	**0.041**	0.664	**0.043**	0.360
	Superonasal	77.82 ± 7.79	81.75 ± 6.65	83.45 ± 8.59	**0.003**	0.054	**0.002**	0.553
RNFL	Average	91.24 ± 9.34	94.81 ± 9.11	93.40 ± 9.07	0.170	0.184	0.768	1.000
	Superior	116.68 ± 12.42	117.44 ± 18.29	118.23 ± 15.97	0.894	1.000	1.000	1.000
	Temporal	66.53 ± 12.04	70.95 ± 9.84	68.18 ± 10.89	0.101	0.146	1.000	0.361
	Inferior	116.62 ± 17.45	120.22 ± 15.13	118.26 ± 17.09	0.543	0.874	1.000	1.000
	Nasal	66.38 ± 8.07	70.18 ± 8.75	68.48 ± 8.55	0.097	0.101	0.716	0.694

Group A, fellow eyes of choroidal neovascularization patients; Group B, fellow eyes of polypoidal choroidal vasculopathy patients; Group C, control group.

Ganglion cell-inner plexiform layer, GC-IPL; retinal nerve fiber layer, RNFL; SD, standard deviation.

*P-value from one-way analysis of variance among the three groups. SD.

^†^P-value from post hoc test (Bonferroni) between Groups A and B.

^‡^P-value from post hoc test (Bonferroni) between Groups A and C.

^§^P-value from post hoc test (Bonferroni) between Groups B and C.

ANCOVA was performed by adjusting sex, CMT, disc area, and cup volume among the three groups. The estimated average GC-IPL thicknesses in Groups A–C after compensating for covariants were 77.60, 79.83, and 80.90 μm, respectively (p = 0.042; Table 3). Post hoc analyses revealed that differences were only significant between Groups A and C (p = 0.039). The estimated average RNFL thickness did not differ significantly among groups (p = 0.107).

**Table 3:** Average GC-IPL and RNFL thicknesses estimated after adjusting for covariants.

	Group A (n = 34)	Group B (n = 73)	Group C (n = 73)	p-value*	p-value^†^	p-value^‡^	p-value^§^
Average GC-IPL thickness (estimated range; μm)	77.60 (75.44–79.71)	79.83 (78.33–81.34)	80.90 (79.41–82.40)	**0.042**	0.294	**0.039**	1.000
Average RNFL thickness (estimated range; μm)	90.90 (87.94–93.86)	94.82 (92.76–96.87)	93.55 (91.50–95.59)	0.107	0.105	0.448	1.000

Group A, fellow eyes of choroidal neovascularization patients; Group B, fellow eyes of polypoidal choroidal vasculopathy patients; Group C, control group.

*P-value from analysis of covariance among the three groups, with adjustments for sex, central macular thickness, disc area, and cup volume.

^†^P-value from post hoc test (Bonferroni) between Groups A and B.

^‡^P-value from post hoc test (Bonferroni) between Groups A and C.

^§^P-value from post hoc test (Bonferroni) between Groups B and C.

### Determination of factors associated with GC-IPL thickness

**Fig 2:**
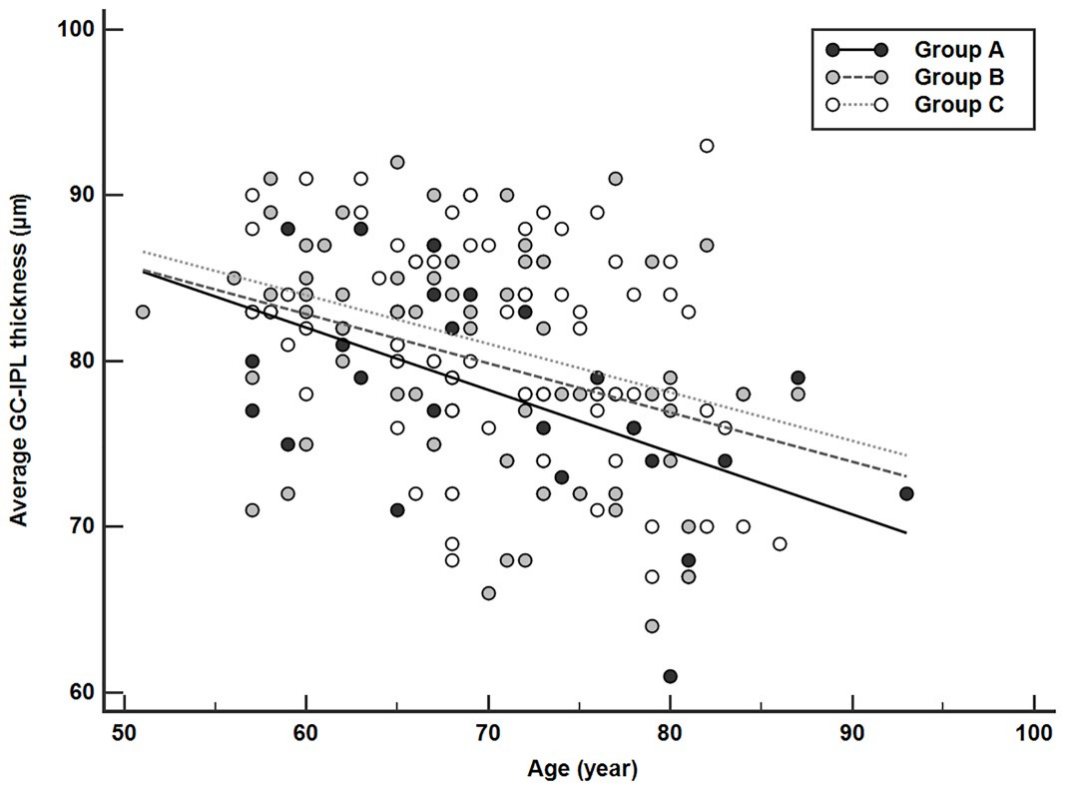
Scatter plot and results of linear regression analysis associating average GC-IPL thickness and age for the fellow eyes of Groups A, B, and C (control). The equations fitted to the regression lines for Groups A–C were: y = –0.405x + 104.504 (p = 0.001), y = –0.297x + 100.608 (p = 0.002), and y = –0.292x + 101.522 (p = 0.003), respectively.

Univariate linear regression analyses revealed that age (p <0.001), BCVA (p = 0.011), CMT (p <0.001), and tCNV (p = 0.008) were associated with average GC-IPL thickness (Table 4). Multivariate linear regression analyses that included four variables from the univariate linear regression analyses showed that age (β = –0.253 ± 0.061, p <0.001), CMT (β = 0.078 ± 0.022, p <0.001), and tCNV (β = –2.864 ± 1.138, p = 0.013) were significant factors. Age was significantly correlated with average GC-IPL thickness in Groups A–C, with slopes of –0.405 (p = 0.001), –0.297 (p = 0.002), and –0.292 (p = 0.003), respectively (Fig 2).

**Table 4:** Results of univariate and multivariate linear regression analyses to evaluate factors affecting GC-IPL thickness.

	Univariate analysis			Multivariate analysis		
Factors	r	β ± SE	p-value	β ± SE	Partial r	p-value
Age	0.342	–0.291 ± 0.060	**<0.001**	–0.253 ± 0.061	**–0.299**	**<0.001**
Sex (0 = male, 1 = female)	0.063	–0.860 ± 1.019	0.400			
BCVA	0.188	34.117 ± 13.334	**0.011**	6.150 ± 12.841	–0.015	0.633
Spherical equivalent	0.059	0.218 ± 0.277	0.434			
Intraocular pressure	0.030	0.078 ± 0.195	0.689			
Axial length	0.076	–0.552 ± 0.556	0.322			
Central macular thickness	0.295	0.094 ± 0.023	**<0.001**	0.078 ± 0.022	**0.194**	**<0.001**
Rim area	0.037	0.026 ± 0.052	0.618			
Disc area	0.130	2.521 ± 1.440	0.082			
C/D ratio	0.056	–2.374 ± 3.184	0.457			
Cup volume	0.008	0.341 ± 3.241	0.916			
tCNV	0.198	–3.398 ± 1.258	**0.008**	–2.864 ± 1.138	**–0.156**	**0.013**
PCV	0.031	0.423 ± 1.023	0.680			

SE, standard error; BCVA, best-corrected visual acuity; C/D, cup to disc; tCNV, typical choroidal neovascularization; PCV, polypoidal choroidal vasculopathy.

r represents the zero-order correlation; β represents the standardized regression weights; Partial r represents partial correlation coefficient which means the association between two variables after adjusting the effects of additional variables.

Boldface numbers indicate statistically significant associations (p <0.05).

## Discussion

Studies on GC-IPL thickness have reported observations of various ocular disorders, including glaucoma, neuro-ophthalmic diseases, and macular diseases [21–24]. Approximately half of the retinal ganglion cells are concentrated in the macula [25, 26]. Measurement of these large cell bodies in the macular ganglion cell complex has therefore been useful for the detection of many ocular disorders [21–24, 27, 28].

Studies of the inner retinal layers in AMD patients have been reported even before the development of OCT [29–31]. Clarke et al. [31] detected almost complete loss of macular granule cell layer neurons in AMD patients. With the development of OCT, numerous studies have evaluated GC-IPL and RNFL thicknesses in AMD patients. Many studies showed that ganglion cell damage was significantly associated with both dry and exudative AMD, whereas there has been controversy regarding the significance of RNFL thickness [5–9, 32].

There are several mechanisms that can explain thinning of the GC-IPL in AMD patients. First, apoptosis of ganglion cells may be caused by transneuronal degeneration, which is induced from chronically reduced input to the inner retinal layer secondary to photoreceptor damage [10, 33–35]. Second, longstanding outer retinal degeneration in AMD patients may lead to vascular abnormalities in the inner retinal layers, which can affect the thickness of the GC-IPL, with reduced blood flow as identified by OCT angiography [12, 36]. Toto et al. [36] separately analyzed the severity of AMD in individual patients and reported that as dry AMD progressed, vascular abnormalities were induced from the outer to inner retinal layers. In their study, deep vessel density decreased significantly in both early and intermediate dry AMD patients compared to normal controls.

In the present study, the GC-IPL was significantly thinner in the fellow eyes of tCNV patients than in those of normal controls, but no significant differences were found between the fellow eyes of tCNV and PCV patients or PCV patients and controls. Additionally, soft drusen and SDD were found in 82.4% and 29.4% of the fellow eyes of tCNV patients, respectively, and these values were higher than those observed for the fellow eyes of PCV patients, which were 6.8% and 4.1%, respectively. Previous studies have reported that the prevalence of SDD in the fellow eyes of unilateral neovascular AMD patients was 19.4%–41% [37–40], which was consistent with the results of the present study.

The retinal and choroidal vascular characteristics in the fellow eyes of AMD patients showed differences according to the type of wet AMD. Abdolrahimzadeh et al. [41] recently reported that the GC-IPL was thinner in early AMD patients than in healthy controls and significantly thinner in eyes with SDD in respect to eyes with drusen alone. They speculated that SDD was associated with choroidal hypoperfusion [42, 43], which may have induced GC-IPL thinning. In addition, a previous study reported that microvascular perfusion as observed using OCT angiography was decreased further in the fellow eyes of unilateral classic AMD patients compared to unilateral PCV patients [44]. Soft drusen and SDD are uncommon in both unilateral PCV patients and their fellow eyes [45, 46]. Although outer retinal and choroidal abnormalities are commonly found in the fellow eyes of PCV patients [44], their effects on the inner retinal layer are thought to be minimal. Based on these observations, it is assumed that the presence of soft drusen and SDD is an important variable affecting the inner retinal layer.

Lee et al. [6] reported a 5.6% reduction in peripapillary RNFL thickness in dry AMD patients compared to that of the control group; mostly in the temporal sector, whereas no significant reduction in peripapillary RNFL thickness was observed in other studies [5, 8, 32]. On the other hand, Yuda et al. [47] also reported that there was no difference in peripapillary RNFL thickness between disease eyes and fellow eyes in the patients with unilateral exudative AMD, which is consistent with our study. The reason for these results could be that the peripapillary RNFL reflected the entire retina, compared to the macula or GC-IPL thickness, which reflected only the macular area. Alternatively, there is the possibility that ganglion cell damage was not severe enough to cause changes in the RNFL.

Previous studies have reported aging effects on GC-IPL thickness in normal subjects [48–50]. More specifically, a prospective longitudinal study reported that the age-related reduction rate of the average GC-IPL thickness was –0.318 μm/year in normal eyes [50]. In the present study, age-related GC-IPL reductions in the fellow eyes of tCNV (–0.405 μm/year; linear regression analysis) patients were slightly greater than in the other two groups (fellow eyes of PCV patients: –0.297 μm/year, normal controls: –0.292 μm/year; linear regression analysis). This study is a cross-sectional study, and the age-related reduction rates obtained through linear regression may not be accurate. Therefore, it is difficult to directly compare these values with previous studies. However, the tCNV group had a higher reduction rate than the other two groups, and unilateral tCNV was found to be significantly associated with the GC-IPL thickness of fellow eyes using multivariate linear regression analysis, suggesting that unilateral tCNV is an important factor to be considered in the analysis of the GC-IPL.

Our study had some limitations. First, it had a retrospective design, which might have involved selection bias. Second, because tCNV is a less common subtype of exudative AMD than PCV in the Asian population, the number of patients with unilateral tCNV was small. Third, patients with comorbidities such as diabetes mellitus and hypertension were also included in the study. It has been reported that diabetes or hypertension affects GC-IPL and RNFL, and this change can occur even without retinopathy [21, 22, 51, 52]. However, there were no statistically significant differences among the three groups concerning diabetes and hypertension, as presented in Table 1, so it is considered that the effect of diabetes and hypertension would be small. Despite these limitations, the present study could report the inner retinal changes in the fellow eyes of unilateral exudative AMD according to the subtype of AMD, and we also found the factors affecting the inner retinal change, and these results would be helpful to physicians. Additional well-designed longitudinal studies will therefore be needed to investigate differences in the GC-IPL and RNFL using a larger number of patients.

In conclusion, the GC-IPL in the fellow eyes of tCNV patients was thinner than that of the normal control subjects, but no significant differences were found when the fellow eyes were compared between tCNV and PCV patients or PCV patients and controls. In addition, RNFL thickness did not differ among the three groups. In the fellow eyes of exudative AMD patients, drusen were thought to have a significant effect on the ganglion cell layer. The inner retinal layer of fellow eyes showed different characteristics depending on the subtype of AMD, and these results may be helpful in understanding the changes in the inner retinal layer in exudative AMD.

## Supporting information

S1 Data (XLSX)
